# Comparing Potential Drug–Drug Interactions in Companion Animal Medications Using Two Electronic Databases

**DOI:** 10.3390/vetsci8040060

**Published:** 2021-04-08

**Authors:** Tussapon Boonyarattanasoonthorn, Phisit Khemawoot, Anusak Kijtawornrat

**Affiliations:** 1 Department of Physiology, Faculty of Veterinary Science, Chulalongkorn University, Pathumwan, Bangkok 10330, Thailand; tussapon.b@chula.ac.th; 2 Chakri Naruebodindra Medical Institute, Faculty of Medicine Ramathibodi Hospital, Mahidol University, Bang Phli, Samutprakarn 10540, Thailand; 3 Preclinical Pharmacokinetics and Interspecies Scaling for Drug Development Research Unit, Chulalongkorn University, Pathumwan, Bangkok 10330, Thailand

**Keywords:** drug–drug interactions, electronic drug databases, companion animal medications

## Abstract

Multiple-drug prescriptions can cause drug–drug interactions (DDIs), which increase risks associated with healthcare in veterinary medicine. Moreover, many human medicines are used in canine patients under the responsibility of veterinarians and may cause severe problems due to off-label use. Currently, many electronic databases are being used as tools for potential DDI prediction, for example, Micromedex and Drugs.com, which may benefit the prediction of potential DDIs for drugs used in canine. The purpose of this study was to examine different abilities for the identification of potential DDIs in companion animal medicine, especially in canine patients, by Micromedex and Drugs.com. Micromedex showed 429 pairs of potential DDIs, while Drugs.com showed 842 pairs of potential DDIs. The analysis comparing results between the two databases showed 139 pairs (12.28%) with the same severity and 993 pairs (87.72%) with different severities. The major mechanisms of contraindicated and major potential DDIs were cytochrome P450 induction–inhibition and QT interval prolongation. Veterinarians should interpret potential DDIs from several databases with caution and keep in mind that the results might not be reliable due to differences in sensitivity to drugs, drug-metabolizing enzymes, and elimination pathway between animals and humans.

## 1. Introduction

Multiple-drug prescriptions for the treatment of diseases and complications usually occur in humans and animals [1,2,3,4]. One category of adverse drug reactions (ADRs) is defined as drug–drug interactions (DDIs), in which one drug interferes with another. In particular, healthcare providers combine drugs for synergistic action and therapeutic benefit, but toxicity or adverse events may also be possible [5,6,7]. DDIs can lead to drug toxicity or decreased therapeutic effect, resulting in an increase in morbidity and mortality [8,9,10,11,12]. The degree of severity of DDIs is categorized as follows: contraindicated, major, moderate, minor, and none [13,14]. A serious concern is focusing on the contraindicated and major severities when dispensing drugs to patients as well as animals. Recently, attention has shifted to developing online databases for detecting potential DDIs in animals. Online DDI databases have two major types: open-access resources and subscription databases [15,16,17,18]. In general, animal owners usually select open-access databases, for example, Drugs.com, to access potential DDI identification. Conversely, healthcare providers prefer using a subscription database to identify potential DDIs, for example, Micromedex. Interestingly, these two databases have different features [19] and show different results in identifying potential DDIs between prescribed drugs for oral cancer treatment [20]. Additionally, in veterinary medicine, multidrug therapy is commonly used for the treatment of animals [21], and many medicines prescribed for humans are used for animals off-label. Generally, human medicines are tested in animals to clarify efficacy and safety, which may imply that these drugs can also be used in animals, especially dogs, a major species for pharmacokinetic tests during drug development. However, few studies focus on the competency of databases to detect potential DDIs for the management of complicated diseases in animals, for example, cardiovascular diseases, urinary diseases, metabolic diseases, skin diseases, and cancers. These diseases require multiple-drug use and might result in DDIs in sick animals. This study aimed to investigate the differences in the performances of DDI databases for identifying potential DDIs with complicated treatments in animals, such as dilated cardiomyopathy, heart valve regurgitation, urinary tract infections, renal failure, diabetes, hypothyroidism, atopic dermatitis, pyotraumatic dermatitis, lymphoma, and hemangiosarcoma, particularly in dogs, which mostly attend animal hospitals.

## 2. Materials and Methods

### 2.1. Drug Selection

**Figure 1:**
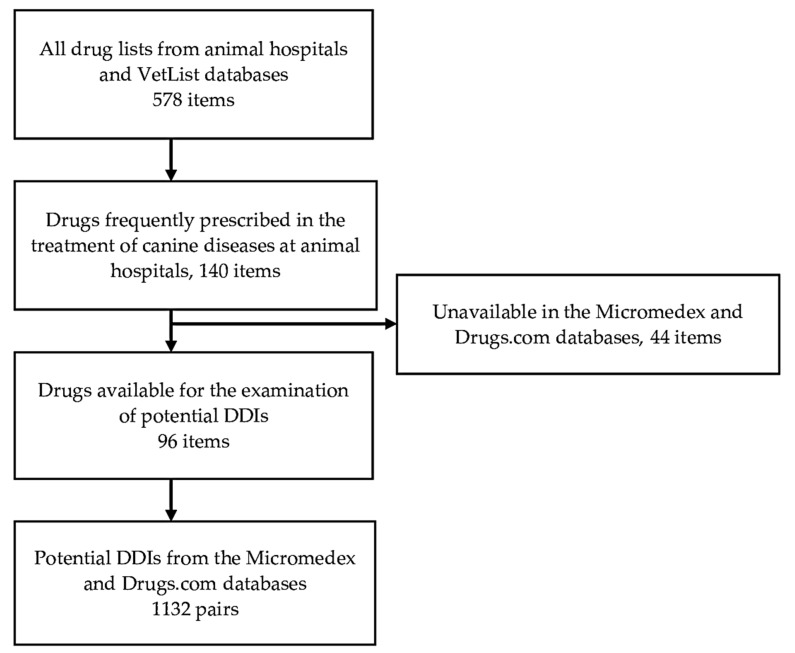
Procedure for drug selection. DDIs: drug–drug interactions.

A list of 578 drugs was taken from those used in animal hospitals located in Thailand and from the VetList database, where all the available animal drugs are listed, accessed on 9 January 2020 [22]. From the total, 140 drugs were selected as frequently prescribed by clinical veterinarians for the treatment of canine diseases in animal hospitals, such as dilated cardiomyopathy, kidney failure, obesity, acute moist dermatitis, and hemangiosarcoma. Remarkably, Micromedex and Drugs.com could not recognize 44 of these drugs due to lack of information in their databases, so those remaining were used for this analysis, as shown in Figure 1 and Table 1. The unrecognized 44 items were aditroprim, afoxolaner, avoparcin, baquiloprim, carprofen, clomocycline, danofloxacin, demethylchlortetracycline, deracoxib, difloxacin, enrofloxacin, eprinomectin, fipronil, firocoxib, glutathione, ibafloxacin, imidacloprid, imidapril, ivermectin, levosimendan, lymecycline, marbofloxacin, methacycline, methoprene, milbemycin oxime, moxidectin, oclacitinib, orbifloxacin, ormetoprim, pimobendan, pirlimycin, pradofloxacin, rolitetracycline, samylin, spiramycin, sulfadimethoxine, sulfadoxine, sulfamethazine, teicoplanin, tepoxalin, tilmicosin, tulathromycin, tylosin, and virginiamycin.

### 2.2. Drug Interaction Database Selection

The subscription drug interaction database Micromedex (Truven Health Analytics, Ann Arbor, MI, USA) was selected for the analysis of potential DDI on the basis of local availability through Chulalongkorn University. The open-access drug interaction database Drugs.com (Drugsite Trust, Auckland, New Zealand) was used because this database is well known and easy to use. The results of DDI from the two databases provided the same information as follows: severity levels, probable mechanism, clinical management, literature, and references. However, Micromedex provided more supportive information about documentation levels and the onset of the interaction.

### 2.3. DDI Categorization

For every selected drug query posed on the databases, the two drug interaction databases categorized potential DDIs with a few different formats and explanations. Micromedex has five severity categories to identify potential DDIs: contraindicated, major, moderate, minor, and unknown. Drugs.com has no category for contraindicated. Interestingly, only Micromedex categorized the documentation level for the detected potential DDIs, at three levels: excellent—the interaction has been clearly verified from controlled studies; good—the interaction is strongly suspected but lacks well-controlled studies; and fair—availability of documentation is poor, but the interaction is suspected to exist on the basis of pharmacological considerations, or a pharmacologically related drug provides good documentation. All results of the potential DDIs in this study were obtained from searches in the two databases and gathered in January 2020.

### 2.4. Data Analysis

Fleiss’ kappa index was used to analyze the agreement in the category of potential DDIs provided by the two drug interaction databases. The kappa value was interpreted by following a guideline that classifies the level of agreement as follows: <0.00, poor agreement; 0.00–0.20, slight agreement; 0.21–0.40, fair agreement; 0.41–0.60, moderate agreement; 0.61–0.80, substantial agreement; and 0.81–1.00, almost perfect agreement [23]. All Fleiss’ kappa calculations were conducted in SPSS for Windows version 22.0 (SPSS Inc., Chicago, IL, USA).

## 3. Results

**Figure 2:**
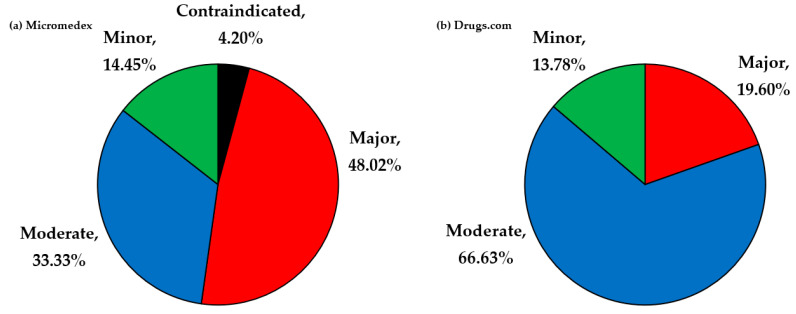
Collation result of the potential DDIs between the two databases: (**a**) Micromedex; (**b**) Drugs.com.

**Figure 3:**
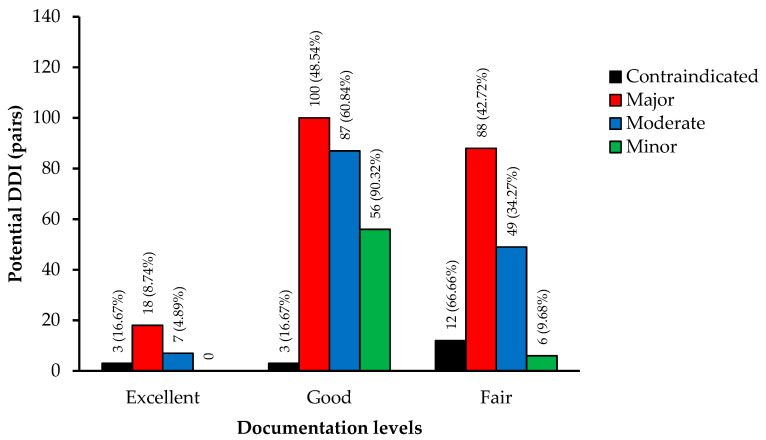
Result of the potential DDIs at each documentation level of Micromedex.

From the 96 drugs used in this study, 1132 unduplicated pairs were found by the selected databases as potential DDIs. Micromedex identified 429 pairs of potential DDIs, and Drugs.com identified 842 pairs of potential DDIs. Figure 2 exhibits the classification of severity for the 429 pairs identified by Micromedex as contraindicated in 18 pairs, major in 206 pairs, moderate in 143 pairs, and minor in 62 pairs. Meanwhile, Drugs.com classified 842 pairs of potential DDIs as 165 pairs in major degree, 561 pairs in moderate degree, and 116 pairs in minor degree. Figure 3 demonstrates the documentation rating in each severity degree of potential DDIs identified by Micromedex, for which the summation of excellent and good scientific evidence was 63.87% (274/429).

After comparing all of the potential DDI results analyzed by the two databases, 139 pairs (12.28%) showed the same severity, while 993 pairs (87.72%) of the results showed a difference in severity. From all of the results, contraindications and major DDIs identified by Micromedex and major DDIs reported by Drugs.com were selected for the determination of the type of mechanism of each potential DDI report, as shown in Supplementary Table S1. Among the 86 pairs of significant potential DDIs, 15 pairs were at the contraindication degree reported by Micromedex and classified at the major degree by Drugs.com. The remaining 71 pairs were reported at the major degree by both databases.

In terms of mechanism of the significant drug pairs in potential DDIs examined by the two databases, 51% (44/86) were pharmacokinetics (PK)-based, 34% (29/86) were pharmacodynamics (PD)-based, and 15% (13/86) were PK–PD-based. The majority of PK-based DDIs involved cytochrome P450 (CYP) induction and inhibition, while PD-based DDIs caused QT prolongation and potassium retention. We also found some conflict between the results of the two databases, in which one database reported potential DDIs as major but the other one reported them as minor or not DDIs. Regarding the dissimilar results as shown in Table 2, 32 pairs were identified by Micromedex as major DDIs but only minor or not DDIs by Drugs.com. Conversely, 53 pairs were specified as major DDIs by Drugs.com, while Micromedex identified these as not DDIs. The agreement in category of potential DDIs provided by the two drug interaction databases as defined by Fleiss’ kappa value was 0.304 (95% CI, 0.206 to 0.402, *p* < 0.001). The kappa value was considered to be a fair agreement between the two databases.

## 4. Discussion

The two databases used in our study could recognize animal medicines less frequently than human medicines. This may be because the medicines are used only in animals. However, Drugs.com included a list of veterinary products that cover many animal species and provided useful information, for example, dosage, administration, precautions, adverse reactions, and especially drug interactions. Therefore, veterinarians should take advantage of this drug database for searching for drug information and as a source for reference documents for their veterinary patients. The potential DDI results from the drug lists were different for the two electronic databases. In this study, the results from Drugs.com exhibited a higher number of potential DDIs than Micromedex by nearly two times. This result was correlated with that of the study of Suriyapakorn et al., who found that Drugs.com provided more sensitivity than the other database for screening DDIs in metabolic syndrome medications [24]. The reason for the high sensitivity of Drugs.com in identifying DDIs may be the use of databases from many providers to analyze data that are contained in Micromedex.

There are many combinations of common drugs that could cause potential DDIs in animals. It has been reported that the use of phenobarbital with phenytoin or quinidine to treat epilepsy in dogs increased the intrinsic clearances of phenytoin and quinidine. Coadministration of ketoconazole and nifedipine in dogs demonstrated twofold increase in the oral bioavailability of nifedipine [25]. Moreover, Intorre et al. [26] reported as a DDI the administration of fluoroquinolones together with theophylline in beagle dogs since an increasing theophylline concentration in the steady state was observed. These combinations could be a cause of potential suffering to animals by altering drug metabolism and excretion. The combination of drugs prescribed to treat canine atopic dermatitis was identified as a potential DDI, for instance, a coprescription of ketoconazole with cyclosporine has been suggested, which could reduce the therapeutic cost and is convenient to use. This combination appears to provide greater clinical benefits for treatment than disadvantages. Nevertheless, an excessive number of alerts regarding potential DDI lacking supporting information could cause wearying among veterinarians as well as animal owners. Many healthcare providers dislike using drug interaction databases [27] for several reasons, including alert fatigue [28,29], workflow disturbance [30], and belief that there is no clinical significance related to most DDI alerts [31]. Ideally, an applicable drug interaction database should have both high sensitivity in identifying significant interactions and high specificity in excluding insignificant interactions. As a result, veterinarians should use more than one DDI reference for reaching the best final answer when identifying potential DDIs [32]. Apart from using several DDI databases, veterinarians should beware of some results from the databases that may be unreliable owing to differences in sensitivity to drugs and drug-metabolizing enzymes among species to preclude animals from adverse events and minimize liability.

In multiple-drug prescriptions, drug dosages usually have a relationship with drug interactions. For example, giving high doses of some drugs may cause interactions, but if they are used at lower doses, the possibility of a DDI may decrease. Ideally, the DDI database should be able to overlook an interaction if the given drugs are at doses that will not likely develop into a DDI [33,34], which the two databases in this study were not able to do. Therefore, the input of dosage should be added to databases so veterinarians can select the doses of drugs under consideration. Interestingly, Micromedex and Drugs.com provide detailed information differently. Micromedex adds more information on allergy interaction, alcohol interaction, lab interaction, pregnancy interaction, and lactation interaction; Drugs.com provides more information only on food interactions and presents results in two categories: consumer and professional. The diversity of information from these two databases gives many benefits to increase the confidence of veterinarians when many health conditions are discussed with their clients.

For the determination of the mechanisms of potential DDIs at contraindicated and major severity levels identified by the two databases, PK-based was the main mechanism of DDI, followed by PD-based and PK–PD-based. The PK-based DDI causes a change of drug concentration in plasma or at the targeted organ by altering the absorption, distribution, metabolism, and elimination. In this study, CYP enzyme inhibition was the main result of PK-based DDI, which leads to the accumulation of coadministered drugs. For example, enrofloxacin, ciprofloxacin, and orbifloxacin inhibit CYP1A activities and decrease the clearance of theophylline, which has been reported in canines, resulting in fatal adverse drug reaction via drug toxicity [26,35]. PD-based DDI is caused by one drug interfering with another drug at the target site. The main result of PD-based DDI in our study was QT prolongation, which may lead to irregular heart rhythm and sudden death. For example, the coadministration of erythromycin with sotalol has been studied in canines and may result in an additive QT prolongation [36]. As a result, veterinarians should truly understand these DDI mechanisms to carefully prescribe multiple drugs and monitor for ADRs properly.

The limitations of this study are described as follows: first, two drug databases were used for the evaluation, so future studies should include additional databases of both subscription and open-access types, such as Lexicomp and Epocrates Free, respectively. Changing of the drug list was also one of our concerns; each year new drugs are developed, while old drugs disappear. The drug list from the animal hospitals used in this study was gathered in the first quarter of 2020, so it might have changed at any time. The updating frequency of databases for their potential DDI reports might affect the results of the analysis. The potential DDI result produced by the updated version of Micromedex and Drugs.com at different time points might give different outcomes from our study, performed in the first quarter of 2020. Additionally, the two databases have no data on some drugs used only in animals, resulting in incomplete potential DDI analysis. Hence, some differences may occur once all drugs are added to the databases or the databases specifically developed for animals. Finally, the results of all probable mechanisms of action in our study referred to humans, which might differ from those for canines due to dissimilar physiologies and drug-metabolizing enzyme systems [37]. However, similar isoforms of the metabolizing enzymes have also been found in canines and other species, which suggests that serious DDIs related to CYP enzymes can occur in canines. Therefore, more study of potential DDIs in animals is recommended to develop veterinary-specific drug databases, improve medical care, and decrease the possibility of DDIs as a result of multidrug therapy in animals.

## 5. Conclusions

Drug interaction databases showed highly variable performances in assessing the DDIs of veterinary drugs. Open-access resources, such as Drugs.com, could detect more potential DDIs. However, Micromedex, a subscription database, provided more supportive information and special features. Although these databases were not specifically developed for use with animals, veterinarians can take the big advantage of the databases in terms of promptness to initially check potential DDIs. Additionally, the thorough judgment of veterinarians should be used to determine appropriate treatments for animals and avoid potential DDIs by using several databases for data evaluation. Hopefully, our study may accelerate the development of specific DDI databases for animals in the near future.

## Figures and Tables

**Table 1:** List of drugs used for the potential DDI analysis.

Drug Class	Drug Groups	Drug Lists
Analgesics	Nonopioid	Meloxicam
Anthelminthics	N/A	Praziquantel
Antiarrhythmics	Antiarrhythmic agent class I	Flecainide Lidocaine Mexiletine Procainamide
	Antiarrhythmic agent class III	Amiodarone Dronedarone Sotalol
	Antiarrhythmic agent class IV	Diltiazem
	Beta-blockers	Atenolol Esmolol Metoprolol
	Cardiac glycosides	Digoxin
Antimicrobials	Aminoglycosides	Amikacin Gentamycin Kanamycin Streptomycin Tobramycin
	Carbapenems	Imipenem/Cilastatin
	Cephalosporins	Cefamandole Cefotaxime Cephalexin Cephalothin Ceftriaxone
	Chloramphenicols	Chloramphenicol
	Fluoroquinolones	Ciprofloxacin
	Glycopeptides	Vancomycin
	Lincosamides	Clindamycin Lincomycin
	Macrolides	Erythromycin
	Monobactams	Aztreonam
	Penicillinase resistant penicillins	Methicillin
	Penicillins	Amoxicillin/Clavulanate Amoxicillin Ampicillin Carbenicillin Nafcillin Oxacillin Penicillin G Penicillin V
	Polymyxins	Polymyxin B Polymyxin E
	Rifamycins	Rifabutin Rifampin Rifapentine
	Sulfonamides	Sulfadiazine Sulfamethoxazole
	Tetracyclines	Chlortetracycline Doxycycline Minocycline Oxytetracycline
	Trimethoprim	Trimethoprim
Anticonvulsants	Barbiturates	Phenobarbital
	Hydantoin	Phenytoin
	Benzodiazepine	Diazepam
	Miscellaneous	Gabapentin Pregabalin
Antifungal agents	Azole derivatives	Itraconazole
	Imidazole derivatives	Ketoconazole Miconazole
Antihistamines	H_1_ receptor antagonists	Cetirizine Chlorpheniramine
Antihypertensive drugs	Angiotensin-converting enzyme inhibitors	Benazepril Enalapril Ramipril
	Beta-blockers	Carvedilol
	Calcium channel blockers	Amlodipine Felodipine Isradipine Nifedipine Nisoldipine Verapamil Nicardipine
Antitussives	N/A	Dextromethorphan
Bronchodilators	Beta-2 receptor agonists	Salmeterol Terbutaline Salbutamol
	Phosphodiesterase inhibitors	Aminophylline Theophylline
Corticosteroids	Systemics	Prednisolone
	Topicals	Fluticasone
Diuretics	Loop diuretics	Furosemide Torsemide
	Potassium-sparing diuretics	Spironolactone
Hormones	Thyroid products	Levothyroxine
Herbal products	N/A	Aloe vera
Immunosuppressants	Calcineurin inhibitors	Cyclosporine
Mucolytic agents	N/A	Acetylcysteine
Expectorants	N/A	Guaifenesin
Vasodilating agents	Phosphodiesterase-5 enzyme inhibitors	Sildenafil
Miscellaneous	Amino acid supplements	Methionine
	Antioxidants	Alpha-lipoic acid
	Antiseptics	Chlorhexidine
	Liver supplements	S-adenosylmethionine
	Vitamin like substances	Coenzyme Q10
N/A, not available		

**Table 2:** Different results between the two databases in the identification of potential DDIs.

Micromedex	Drugs.com	List of Drugs Paired
Major	Minor	Amiodarone—Sulfamethoxazole Digoxin—Gentamicin Digoxin—Spironolactone Digoxin—Trimethoprim Erythromycin—Sulfamethoxazole Flecainide—Trimethoprim Lidocaine—Phenytoin Procainamide—Sulfamethoxazole Sotalol—Sulfamethoxazole
Major	None	Amiodarone—Trimethoprim Amlodipine—Digoxin Amoxicillin—Chlortetracycline Amoxicillin/Clavulanate—Chlortetracycline Ampicillin—Chlortetracycline Chlortetracycline—Methicillin Chlortetracycline—Nafcillin Chlortetracycline—Oxacillin Chlortetracycline—Penicillin G Chlortetracycline—Penicillin V Digoxin—Felodipine Digoxin—Isradipine Digoxin—Meloxicam Digoxin—Nicardipine Erythromycin—Trimethoprim Flecainide—Lidocaine Flecainide—Trimethoprim Isradipine—Procainamide Isradipine—Sulfamethoxazole Isradipine—Trimethoprim Itraconazole—Sotalol Mexiletine—Sotalol Sotalol—Trimethoprim
None	Major	Albuterol—Carvedilol Amikacin—Polymyxin B Aminophylline—Carvedilol Amiodarone—Furosemide Amiodarone—Nafcillin Amiodarone—Phenobarbital Amiodarone—Rifabutin Amiodarone—Terbutaline Amlodipine—Rifabutin Atenolol—Aminophylline Atenolol—Theophylline Diltiazem—Flecainide Diltiazem—Itraconazole Diltiazem—Rifabutin Erythromycin—Itraconazole Erythromycin—Sildenafil Esmolol—Aminophylline Felodipine—Rifabutin Gentamicin—Polymyxin B Isradipine—Phenobarbital Isradipine—Rifabutin Itraconazole—Amlodipine Itraconazole—Isradipine Itraconazole—Nicardipine Itraconazole—Rifapentine Kanamycin—Polymyxin B Metoprolol—Aminophylline Metoprolol—Theophylline Nicardipine—Phenobarbital Nicardipine—Rifabutin Phenobarbital—Amlodipine Phenobarbital—Nisoldipine Phenytoin—Felodipine Phenytoin—Isradipine Phenytoin—Nicardipine Phenytoin—Amlodipine Procainamide—Terbutaline Rifabutin—Nisoldipine Rifampin—Cefamandole Rifampin—Felodipine Rifampin—Nicardipine Salmeterol—Carvedilol Sotalol—Albuterol Sotalol—Aminophylline Sotalol—Salmeterol Sotalol—Terbutaline Streptomycin—Polymyxin B Terbutaline—Carvedilol Theophylline—Carvedilol Theophylline—Esmolol Theophylline—Sotalol Tobramycin—Polymyxin B Verapamil—Itraconazole

## Data Availability

The data presented in this study are available within the article.

## Supplementary Materials

The following are available online at https://www.mdpi.com/article/10.3390/vetsci8040060/s1: Table S1: Significant drug pairs in potential DDIs examined by the two databases.
