# Supplementary material for: Comparing Potential Drug–Drug Interactions in Companion Animal Medications Using Two Electronic Databases
Source: Vet Sci. 2021 Apr 8;8(4):60. doi: 10.3390/vetsci8040060 (PMC8068153; doi:10.3390/vetsci8040060)
Supplement: Supplementary file 1 [file vetsci-08-00060-s001.pdf]

Supplementary Materials

Table S1. The significant drug pairs in potential DDIs examined by the two databases.

Micromedex	Drugs.com	List of drugs paired	PK-PD	Mechanism details
Contraindication	Major	1. Amiodarone—Dronedarone	PD	Additive QT-interval prolongation
		2. Amiodarone—Ketoconazole	PK	CYP3A inhibition by Ketoconazole
		3. Ciprofloxacin—Dronedarone	PD	Additive QT-interval prolongation
		4. Cyclosporine—Dronedarone	PK	CYP3A inhibition by Cyclosporine
		5. Dronedarone—Erythromycin	PK	CYP3A inhibition by Erythromycin
		6. Dronedarone—Flecainide	PD	Additive QT-interval prolongation
		7. Dronedarone—Itraconazole	PK	CYP3A4 inhibition by Itraconazole
		8. Dronedarone—Ketoconazole	PK	CYP3A inhibition by Ketoconazole
		9. Dronedarone—Procainamide	PD	Additive QT-interval prolongation
		10. Dronedarone—Sotalol	PD	Additive QT-interval prolongation
		11. Felodipine—Itraconazole	PK	CYP3A inhibition by Itraconazole
		12. Felodipine—Ketoconazole	PK	CYP3A inhibition by Ketoconazole
		13. Itraconazole—Nisoldipine	PK	CYP3A inhibition by Itraconazole
		14. Ketoconazole—Nisoldipine	PK	CYP3A inhibition by Ketoconazole
		15. Praziquantel—Rifampin	PK	CYP induction by Rifampin
Major	Major	1. Amikacin—Furosemide	PD	Additive or synergistic toxicity
		2. Aminophylline—Ciprofloxacin	PK	Decreased clearance of Theophylline by Ciprofloxacin
		3. Aminophylline—Mexiletine	PK	Decreased hepatic metabolism
		4. Amiodarone—Ciprofloxacin	PD	Additive effects on QT interval
		5. Amiodarone—Digoxin	PK	P-glycoprotein inhibition by Amiodarone
		6. Amiodarone—Erythromycin	PD, PK	Additive effects on QT prolongation, CYP3A inhibition by Erythromycin
		7. Amiodarone—Flecainide	PD, PK	Antiarrhythmic inhibition by Amiodarone, CYP2D inhibition by Amiodarone
		8. Amiodarone—Itraconazole	PK	CYP3A inhibition by Itraconazole
		9. Amiodarone—	PD	Antiarrhythmic inhibition by

	Procainamide		Amiodarone
10.	Amiodarone—Rifampin	PK	CYP induction by Rifampin
11.	Amiodarone—Sotalol	PD	Additive effects on refractory potential
12.	Amiodarone—Verapamil	PK	CYP3A inhibition by Verapamil
13.	Amiodarone—Diltiazem	PK	CYP3A inhibition by Diltiazem
14.	Amlodipine—Rifampin	PK	CYP induction by Rifampin
15.	Atenolol—Verapamil	PD, PK	Additive cardiovascular effects, decreased metabolism of some beta-blockers by Verapamil
16.	Atenolol—Diltiazem	PD, PK	Additive cardiovascular effects, decreased metabolism of some beta-blockers by Diltiazem
17.	Benazepril—Spironolactone	PD	Increased potassium retention secondary to lowered aldosterone levels
18.	Benazepril—Trimethoprim	PD	Additive effects of hyperkalemia
19.	Carvedilol—Verapamil	PD, PK	Additive cardiovascular effects, decreased metabolism of some beta-blockers by Verapamil
20.	Carvedilol—Diltiazem	PD, PK	Additive cardiovascular effects, decreased metabolism of some beta-blockers by Diltiazem
21.	Ciprofloxacin—Prednisolone	PD	An additive effect of risk for tendon rupture
22.	Ciprofloxacin—Procainamide	PD	Additive effects on the QT interval
23.	Ciprofloxacin—Sotalol	PD	Additive effects on the QT interval
24.	Ciprofloxacin—Theophylline	PK	Decreased clearance of Theophylline, CYP1A inhibition by Ciprofloxacin
25.	Cyclosporine—Itraconazole	PK	CYP3A inhibition by Itraconazole
26.	Cyclosporine—Rifabutin	PK	Cyclosporine metabolism induction by Rifabutin
27.	Cyclosporine—Rifampin	PK, PD	Increased Cyclosporine clearance and decreased systemic bioavailability by Rifampin
28.	Digoxin—Dronedarone	PK	P-glycoprotein inhibition by Dronedarone
29.	Digoxin—Itraconazole	PK	Digoxin metabolism and clearance inhibition by Itraconazole
30.	Enalapril—Spironolactone	PD	Increased potassium retention secondary to lowered aldosterone

			levels
31.	Enalapril—Trimethoprim	PD	Additive effects of hyperkalemia
32.	Erythromycin—Ketoconazole	PK	CYP3A inhibition by Erythromycin and Ketoconazole
33.	Erythromycin—Procainamide	PK	CYP3A inhibition by Erythromycin
34.	Erythromycin—Sotalol	PD	Additive effects on QT prolongation
35.	Erythromycin—Verapamil	PK	CYP3A inhibition by Erythromycin
36.	Erythromycin—Diltiazem	PK	CYP3A-mediated inhibition by Diltiazem
37.	Esmolol—Verapamil	PD, PK	Additive cardiovascular effects, decreased metabolism of some beta-blockers by Verapamil
38.	Esmolol—Diltiazem	PD, PK	Additive cardiovascular effects, decreased metabolism of some beta-blockers by Diltiazem
39.	Flecainide—Procainamide	PD	Additive effects on QT prolongation
40.	Flecainide—Sotalol	PD	Additive effects on refractory potential
41.	Fluticasone—Itraconazole	PK	CYP3A-mediated inhibition by Itraconazole
42.	Fluticasone—Ketoconazole	PK	CYP3A-mediated inhibition by Ketoconazole
43.	Furosemide—Gentamicin	PD	Additive or synergistic toxicity
44.	Furosemide—Kanamycin	PD	Additive or synergistic toxicity
45.	Furosemide—Streptomycin	PD	Additive or synergistic toxicity
46.	Furosemide—Tobramycin	PD	Additive or synergistic toxicity
47.	Itraconazole—Nifedipine	PK	CYP3A inhibition by Itraconazole
48.	Itraconazole—Rifabutin	PK	CYP3A induction by Rifabutin, CYP3A inhibition by Itraconazole
49.	Itraconazole—Rifampin	PK	CYP3A-mediated induction by Rifampin
50.	Itraconazole—Sildenafil	PK	CYP3A inhibition by Itraconazole
51.	Ketoconazole—Procainamide	PD	Additive QT-interval prolongation
52.	Ketoconazole—Rifabutin	PK	CYP3A-mediated inhibition by Ketoconazole, CYP3A-mediated induction by Rifabutin
53.	Ketoconazole—	PK	CYP3A-mediated induction by

	Rifampin		Rifampin
54.	Ketoconazole—Rifapentine	PK	CYP3A induction by Rifapentine
55.	Ketoconazole—Sotalol	PD	Additive effects on the QT interval
56.	Metoprolol—Verapamil	PD, PK	Additive cardiovascular effects, decreased metabolism of some beta-blockers by Verapamil
57.	Metoprolol—Diltiazem	PD, PK	Additive cardiovascular effects, decreased metabolism of some beta-blockers by Diltiazem
58.	Mexiletine—Theophylline	PK	Decreased hepatic metabolism, CYP1A inhibition by Mexiletine
59.	Nifedipine—Phenobarbital	PK	CYP3A induction by Phenobarbital
60.	Nifedipine—Phenytoin	PK	CYP3A induction by Phenytoin
61.	Nifedipine—Rifabutin	PK	CYP3A induction by Rifabutin
62.	Nifedipine—Rifampin	PK	CYP3A induction by Rifampin
63.	Nifedipine—Rifapentine	PK	CYP3A induction by Rifapentine
64.	Procainamide—Sotalol	PD	Additive effects on refractory potential
65.	Ramipril—Spironolactone	PD	Increased potassium retention secondary to lowered aldosterone levels
66.	Ramipril—Trimethoprim	PD	Additive effects of hyperkalemia
67.	Salmeterol—Itraconazole	PK	CYP3A-mediated inhibition by Itraconazole
68.	Salmeterol—Ketoconazole	PK	CYP3A-mediated inhibition by Ketoconazole
69.	Sotalol—Verapamil	PD, PK	Additive cardiovascular effects, decreased metabolism of some beta-blockers by Verapamil
70.	Sotalol—Diltiazem	PD, PK	Additive cardiovascular effects, decreased metabolism of some beta-blockers by Diltiazem
71.	Spironolactone—Trimethoprim	PD	Additive effects of hyperkalemia
